# Unwinding of Medicaid enrollment and increased uninsured emergency department visits in California

**DOI:** 10.1093/haschl/qxaf238

**Published:** 2025-12-22

**Authors:** Nima Khodakarami, Theodoros V Giannouchos, Daniel Marthey, Benjamin Ukert, Joel Segel, Laura Dague

**Affiliations:** Department of Health Policy and Administration, Penn State University, Monaca, PA 15061, United States; Center for Applied Studies in Health Economics, College of Medicine, Penn State University, Hershey, PA 17033, United States; Department of Health Policy and Organization, School of Public Health, the University of Alabama at Birmingham, Birmingham, AL 35294, United States; Center for Outcomes and Effectiveness Research and Education, Heersink School of Medicine, the University of Alabama at Birmingham, Birmingham, AL 35233, United States; Department of Health Policy and Management, School of Public Health, Texas A&M University, College Station, TX 77843, United States; Department of Health Policy and Management, School of Public Health, Texas A&M University, College Station, TX 77843, United States; Department of Health Policy and Administration, Penn State University, University Park, PA 16802, United States; Penn State Cancer Institute, Hershey, PA 17033, United States; Department of Public Health Sciences, Penn State College of Medicine, Hershey, PA 17033, United States; Department of Public Service and Administration, Bush School of Government and Public Service, Texas A&M University, College Station, TX 77845, United States

**Keywords:** Medicaid unwinding, emergency department, insurance coverage, acute care, safety net, rural hospitals, critical access hospitals, uninsured, uncompensated care, hospital ownership, California

## Abstract

**Objective:**

To examine the association between post–pandemic era Medicaid eligibility redeterminations (“unwinding”) and emergency department (ED) payer mix in California.

**Methods:**

We conducted a retrospective secondary analysis of California's quarterly ED visit data (2021–2024) across 281 general acute-care hospitals, using interrupted time-series analysis.

**Results:**

During unwinding, Medicaid ED visits declined by 0.37 percentage points (pp) and uninsured ED visits increased by 0.16 pp per quarter (*P* < .001) relative to the pre-unwinding period. The largest Medicaid declines occurred in investor-owned (0.87 pp, 11.9%) and medium-sized (0.47 pp, 7.4%) hospitals (*P* < .001). In contrast, the largest increases in uninsured visits occurred among hospitals in rural (0.29 pp, 46.7%) and high-poverty (0.24 pp, 7.7%) areas, in addition to small hospitals (0.22 pp, 25.8%) (*P* < .001). Private visits saw an immediate decrease of 0.49 pp (*P* < .001), followed by a continued reduction of 0.15 pp per quarter (*P* < .05), showing flattening of the previously increasing trend. Medicare visits increased by 0.18 pp per quarter (*P* < .001) relative to the pre-unwinding period.

**Conclusion:**

Medicaid unwinding was associated with a decline in Medicaid ED visits and a corresponding increase in uninsured ED visits, with varying impacts across hospital types in California.

Key Points

- Medicaid unwinding in California was associated with a declining trend in Medicaid paid emergency department visits, while the trend in uninsured and Medicare visits relative to pre-unwinding began to increase.
- Smaller and rural hospitals saw the most pronounced increases in uninsured visits, while investor-owned hospitals experienced the largest declines in Medicaid visits relative to pre-unwinding.
- Our findings suggest an increasing burden of uncompensated care cost with implications for hospital profit margins.

## Introduction

Beginning April 1, 2023, states were required to resume determining the eligibility of every Medicaid beneficiary as part of “unwinding” the continuous enrollment provisions of the COVID-19 public health emergency (PHE).^1^ Over the course of the 14 months of redeterminations, approximately 25 million people lost Medicaid coverage, approximately 1 in 4 of total enrollment under temporary provisions, requiring states to extend continuous coverage to all Medicaid enrollees.^2^ A survey of nonelderly adults in certain states found that approximately half those losing Medicaid became uninsured^3^; under typical circumstances, 1 in 5 Medicaid enrollees lose coverage at renewal,^4^ with half of those who lose coverage for any reason remaining uninsured 6 months later.^5^ Limited awareness of the administrative processes, confusion about eligibility and disenrollment timing, and learning about coverage loss only when seeking care contribute to many people remaining uninsured.^3,5-7^

Hospitals faced financial risks from unwinding, as changes in payer mix could lead to higher rates of uncompensated care if many Medicaid patients become and remain uninsured.^8,9^ This may be particularly relevant for emergency departments (EDs), since lower-income patients exhibit disproportionately higher reliance on ED use and federal law requires EDs to provide screening and stabilization regardless of ability to pay.^10-12^ Therefore, identifying the extent and types of hospital EDs most affected by the unwinding could help determine which hospitals are at greatest risk of financial loss due to increases in uncompensated care.

In this study, we estimated the association between unwinding and changes in ED payer mix in California. California makes a compelling case study because it has the nation's largest Medicaid program, Medi-Cal, which had enrolled more than 14 million beneficiaries during the peak of the PHE. The state's unwinding process, which began in July 2023, ultimately disenrolled nearly 2 million individuals from Medicaid, a 13% reduction and the third highest disenrollment after Texas and New York.^13-15^

Using data for more than 44 million ED visits across 281 hospitals, we examined changes in payer mix both overall and by hospital characteristics, with a focus on hospitals that may be most vulnerable to financial strain. Our analysis is among the first empirical evidence of unwinding's impact on payer mix and offers insight into how large-scale Medicaid policy changes translate into the health care delivery system.

## Methods

We conducted a retrospective secondary analysis of ED visits from the first quarter of 2021 through the fourth quarter of 2024 using public data from the California Department of Health Care Access and Information Report Center.^16^ The data provide quarterly reports of outpatient emergency care visits (treated and released the same day) collected from general acute-care hospitals.^17^ For each facility, the number of quarterly ED visits was available by expected payer and their categories (Medi-Cal, private, Medicare, uninsured, other), sex (female, male), race (American Indian or Alaska Native, Asian, Black or African American, Native Hawaiian or other Pacific Islander, White, multiracial, other race), ethnicity (Hispanic or Latino, non-Hispanic), age (0 to 80+ years in 10-year intervals), residency (Californian resident, out of state/international, and unknown/homeless), preferred language spoken (English, not-English), and disposition or discharge information (which we grouped as routine care, ie, home or self-care vs all other).

Primary outcomes were the total quarterly number and share of ED encounters by payer overall and by facility type. Information on facility characteristics was obtained from the California Health and Human Services open data portal.^18^ We categorized facilities by urbanicity (urban/rural), ownership type (nonprofit, investor-owned, and state/local), number of licensed beds (<100 beds, 100–299 beds, ≥300 beds), and location in a county with a poverty rate above the mean California rate (12.5%).^19^ We limited the sample to facilities with complete 4-quarter reports for each year, resulting in a balanced panel of 281 facilities (91% of 309 total).

### Statistical analysis

We compared the volume and share of ED visits stratified by patient characteristics before (2021 quarter [Q] 1–2023 Q2) and during (2023 Q3–2024 Q4) the unwinding period using *t* tests. We also plotted visit volumes overall and by payer over time to visualize trends before and during the unwinding. We used unweighted interrupted time-series (ITS) analysis to examine changes in ED visits by payer type, which allowed us to capture both any immediate-level shift and change in trend reflecting the gradual nature of the unwinding. We adjusted for factors that could influence visit patterns, including the percentage of patient characteristics by facility-quarter (age, race and ethnicity, sex, language, and disposition). Models included facility and quarter fixed effects to control for time-invariant facility characteristics and seasonal variation, respectively, and were estimated with robust SEs, clustered by facility. We performed a structural break analysis to validate the occurring intercept and trend-shift changes (stationary trend break) when the unwinding process began in 2023 Q3. The Harris–Tzavalis test confirmed that our outcomes were stationary.^20^ We used model- and moment-selection criteria (MMSC) corresponding to the Akaike information criterion (AIC) to choose the appropriate number of lags for each outcome, which varied between 6 and 10.^21,22^

We analyzed subgroups by hospital characteristics (rurality, ownership, bed size, and poverty level of hospital counties). Sensitivity analyses included all 309 hospitals to evaluate whether our results were sensitive to the sample selection. All analyses were replicated using weighted estimates accounting for the visit-volume of each facility to evaluate robustness of findings.

## Results

A total of 44.6 million outpatient ED visits across 281 hospitals occurred during the study period. The average volume of quarterly visits at each hospital was 9935.5 (SD = 6818.8) (Appendix Table S1). Medi-Cal was the most common payer, accounting for 42% of visits, followed by private insurance (29%) and Medicare (21%). Uninsured visits represented 6% of all encounters. Most visits were by females (53%), Whites (57%), and non-Hispanics (59%). Nearly all visits were made by California residents (96%) and English speakers (85%), and most resulted in routine discharges to home or self-care (92%) (Appendix Table S1). Most visits occurred at urban hospitals (92%), while nonprofit hospitals accounted for the largest share of visits (69%), followed by investor-owned (17%) and state/local (14%) hospitals (Appendix Table S2).

**Figure 1.**
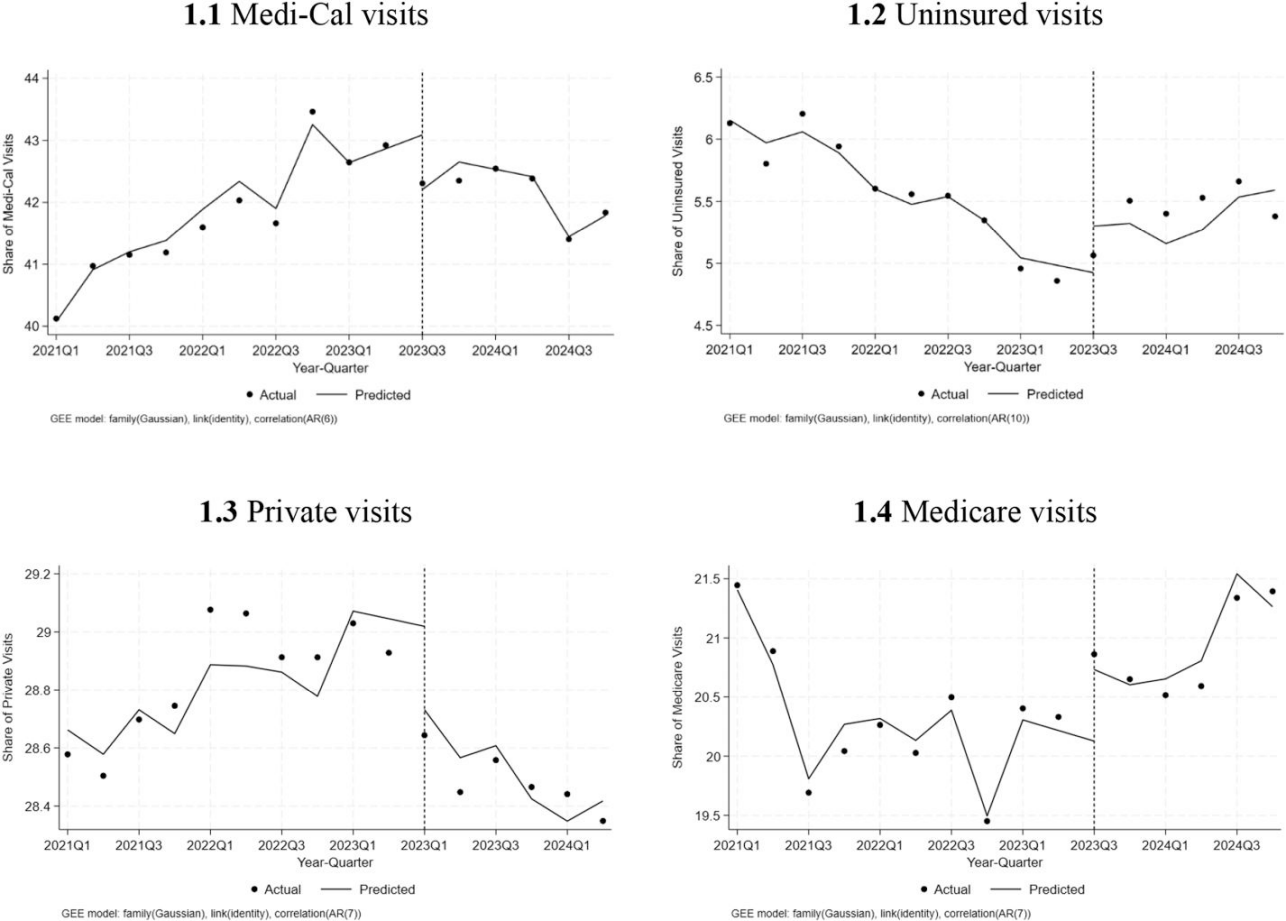
Interrupted time-series analysis of Medicaid and uninsured emergency department visits before and after Medicaid unwinding in California, 2021 Q1–2024 Q4. Abbreviations: AR, autoregressive; GEE, generalized estimating equation; Q, quarter.

The share of Medi-Cal visits grew by 0.19 percentage points (pp) per quarter prior to the unwinding, while the share of uninsured had been decreasing by 0.1 pp (Table 1). Interrupted time-series analysis did not detect an immediate shift in Medi-Cal or uninsured visits at the quarter of unwinding, but indicated a significant trend change, showing a decline of 0.37 pp per quarter (*P* < .001) in Medi-Cal visits and an increase of 0.16 pp per quarter (*P* < .001) in uninsured visits relative to the pre-unwinding period (Table 1, Figure 1). This indicates an approximately 1.6% decrease in Medi-Cal visits and 10.2% increases in uninsured visits relative to the average 2 quarters preceding the unwinding in 2023.

**Table 1.** Association of Medicaid unwinding with quarterly payer mix of outpatient emergency department visits in California (2021–2024): adjusted estimates, balanced sample

	Pre-unwinding trend	Level change after unwinding	Trend change relative to the pre-unwinding trend
Medi-Cal	0.19*** (0.05)	−0.14 (0.23)	−0.37*** (0.08)
Private	0.17*** (0.05)	−0.49*** (0.14)	−0.15* (0.06)
Uninsured	−0.10*** (0.03)	0.21 (0.18)	0.16*** (0.03)
Medicare	−0.10*** (0.02)	0.09 (0.10)	0.18*** (0.05)
Other	−0.05** (0.02)	0.10* (0.04)	0.07** (0.02)

The estimates are for 281 facilities licensed as general acute-care hospitals. Each row presents estimates from a separate interrupted time-series regression model stratified by payer type. Coefficients for trends and level-changes are based on unweighted interrupted time-series estimates. The models were adjusted for patient characteristics (eg, age, race and ethnicity, sex, language, and disposition), facility fixed effects, and quarter fixed effects. Robust SEs clustered at the facility level are shown in parentheses. *P < .05, **P < .01, ***P < .001.

We also observed a significant decline in ED visits covered by private insurance plans during the unwinding period, with an immediate decrease of 0.49 pp (*P* < .001), followed by a continued reduction of 0.15 pp per quarter (*P* < .05) relative to the pre-unwinding trend, showing flattening of the previously increasing trend. The trend in Medicare visits increased by 0.18 pp per quarter (*P* < .001) relative to the pre-unwinding trend (Table 1, Figure 1).

Our ITS estimates from a visit-weighted specification were robust to variation in hospital size (Appendix Table S3). Our findings were further validated for the ITS estimates using all 309 hospitals that reported data at some point in the dataset, suggesting no sample selection bias (Appendix Table S4).

Analyses by hospital types indicated that all hospital types experienced reductions in ED visit Medi-Cal shares (Appendix Table S5). The largest increases in uninsured ED visits were observed among rural hospitals, hospitals in high-poverty counties, and small hospitals (<100 beds), with trend increases of 0.29, 0.24, and 0.22 pp per quarter (*P* < .001), respectively. Other hospital types experienced more moderate and mixed changes.

## Discussion

Our findings suggest that the unwinding was associated with a shift in the ED visit payer mix over time. We found that Medicaid unwinding led to measurable, but modest effects on hospital ED visit payer mix. With unwinding, the upward trend in Medi-Cal–paid ED visit share reversed, and the downward trend in uninsured visit share was also reversed to become an increase. We further found an increase in the Medicare visit share and a stagnation in private ED visits. Although these changes aligned with pre–COVID-19 trends,^23^ they could also reflect substitution effects among dual-eligible or dual-enrolled individuals losing Medicaid coverage,^24-26^ or broader demographic trends.

These changes in California were smaller than those found in some other states, likely due to differences in approaches to the unwinding process and Medicaid eligibility policy as well as the number of affected residents. A study from Texas found much larger immediate shifts, followed by approximately 7 times greater quarterly changes in uninsured visit shares (in pp) than those observed in our study, as well as greater substitution to private insurance coverage.^27^ Several factors may explain these differences. First, California automatically enrolled close to 770 000 Medi-Cal disenrollees into Marketplace silver plans as of December 2024, which could have mitigated coverage gaps, although our data do not show measurable offsetting increases in privately insured visits; perhaps because only approximately 36% of enrollees who were eligible for private coverage confirmed and effectuated their plans.^28^ Second, hospitals with more than 100 beds in California may have participated in Hospital Presumptive Eligibility and offered temporary Medicaid coverage for eligible enrollees.^29^ Finally, many disenrolled individuals may have used less ED care,^30^ or had secondary insurance, such as Medicaid enrollees who became Medicare-eligible during the PHE.

The burden of unwinding was not equally distributed across hospitals. While investor-owned hospitals experienced the largest relative declines in Medi-Cal visits, rural hospitals, hospitals in high-poverty counties, and small hospitals (<100 beds) saw the most pronounced increases in uninsured ED visits. One unobserved, but potentially important, driver might be variation across California in the rates at which those losing Medi-Cal enrolled in different types of coverage or remained uninsured. These findings provide a more nuanced understanding of the differential impact of unwinding across hospital types. These findings suggest that hospitals heavily reliant on Medicaid patients as a primary source of revenue^31-34^ experienced the greatest increases in uninsured ED visits following the unwinding, while the change in uninsured visits was less pronounced among other hospitals. These facilities already face financial pressures due to proposed cuts to disproportionate-share hospital payments and reductions in supplemental payments, and on average, operate on thin or negative margins, restraining their ability to absorb additional uncompensated care.^35^

Based on previous work, ED discharges for both Medicaid and uninsured patients are associated with negative profits, and an increase in the share of uninsured patients could add additional strain to hospital profitability.^36^ In our study, after 6 quarters, the average share of uninsured visits increased by 0.96 pp. This translates into approximately 171 000 additional uninsured visits overall, and approximately $121 million in costs (see Appendix for estimations), assuming an average cost of treat-and-release ED visits for routine discharges of $710 and applying a 40% reimbursement ratio for Medicaid patients compared with uninsured patients.^37,38^ These findings underscore the need for policymakers to weigh the financial impact of newly proposed cuts in Medicaid expenditure on providers.^39,40^

This study is not without limitations. Our findings are limited to outpatient ED visits given the data availability, which represent approximately 90% of all ED visits.^41,42^ Our focus on California may also limit generalizability to other (particularly non-expansion) states, where unwinding effects are likely to be larger, as California took a more proactive approach by automatically enrolling many Medi-Cal disenrollees into Marketplace silver plans and helping disenrollees find coverage.^28^ Finally, the ITS design cannot differentiate between the many co-occurring policy changes during the end of the PHE, so we cannot definitively attribute all changes in trends and levels to Medicaid unwinding.

In summary, the partial shift from Medicaid to uninsured ED visits without offsetting increases in private coverage suggests an increasing burden of uncompensated care cost with a potential impact on hospitals’ profit margins, particularly for smaller and rural hospitals that exhibited the largest increases in uninsured visits.^8^ However, our estimates suggest that the relative compositional changes in payer mix and their financial impact may be limited in California, given that aggregate costs for Medicaid ED visits was $21.4 billion based on the most recent available data from 2021.^37^ Further research across other states is needed to describe the trend in payor mix in outpatient and inpatient settings.

## Supplementary Material

qxaf238_Supplementary_Data

## Data Availability

The datasets were derived from publicly available sources described in the “Methods” section.
