# Supplementary material for: Unwinding of Medicaid enrollment and increased uninsured emergency department visits in California
Source: Health Aff Sch. 2025 Dec 22;4(1):qxaf238. doi: 10.1093/haschl/qxaf238 (PMC12849369; doi:10.1093/haschl/qxaf238)
Supplement: qxaf238_Supplementary_Data [file qxaf238_supplementary_data.zip › Appendix.docx]

**Appendix**

**Table 1A** Descriptive characteristics of ED visits in California, pre- and during Medicaid unwinding

|  | Total | Pre-unwinding | During unwinding | P-value |
| --- | --- | --- | --- | --- |
|  | 2021Q1 to 2024Q4 | 2021Q1 to 2023Q2 | 2023Q3 to 2024Q4 |  |
| Observations | 4,496 | 2,810 | 1,686 |  |
| Total visits | 44,620,104 | 26,595,680 | 18,024,424 |  |
| Quarterly visits per hospital - mean | 9,935.5 (6,818.8) | 9,481.5 (6,540.9) | 10,690.6 (7,196.4) | <0.001 |
| Health insurance, **^a^** % (SD) |  |  |  |  |
| Medi-Cal | 41.9 (18.5) | 41.7 (18.5) | 42.1 (18.6) | 0.526 |
| Private | 28.7 (15.9) | 28.8 (16.0) | 28.4 (15.7) | 0.461 |
| Uninsured | 5.5 (3.1) | 5.5 (3.3) | 5.4 (2.5) | 0.069 |
| Medicare | 20.5 (9.4) | 20.3 (9.2) | 20.8 (9.6) | 0.041 |
| Other payers **^b^** | 3.1 (2.8) | 3.2 (3.1) | 3.0 (2.2) | 0.006 |
| Sex, **^a^** % (SD) |  |  |  |  |
| Male | 46.7 (3.8) | 46.8 (4.1) | 46.6 (3.4) | 0.064 |
| Female | 53.1 (3.9) | 52.9 (4.2) | 53.3 (3.4) | 0.001 |
| Race, **^a^** % (SD) |  |  |  |  |
| White | 57.2 (22.4) | 57.5 (22.4) | 56.6 (22.4) | 0.207 |
| Black or African American | 8.5 (8.7) | 8.6 (9.1) | 8.3 (8.5) | 0.182 |
| American Indian or Alaska Native | 0.82 (1.8) | 0.81 (1.8) | 0.84 (1.8) | 0.619 |
| Asian/ Native Hawaiian/ Pacific Islander | 6.8 (8.3) | 6.6 (8.2) | 7.0 (8.4) | 0.152 |
| Multiracial/ Other | 24.3 (18.2) | 24.0 (18.0) | 24.8 (18.5) | 0.137 |
| Ethnicity, **^a^** % (SD) |  |  |  |  |
| Hispanic | 38.9 (21.4) | 38.1 (21.2) | 40.1 (21.6) | 0.002 |
| Not Hispanic | 58.8 (20.9) | 59.6 (20.8) | 57.5 (20.9) | <0.001 |
| Age groups, **^a^** % (SD) |  |  |  |  |
| 0 to 19 | 20.6 (14.3) | 20.5 (14.3) | 20.8 (14.2) | 0.568 |
| 20 to 39 | 29.5 (7.1) | 29.9 (7.2) | 28.8 (6.8) | <0.001 |
| 40 to 59 | 23.9 (5.0) | 24.1 (5.1) | 23.6 (4.8) | 0.001 |
| 60 and above | 25.7 (9.1) | 25.1 (9.0) | 26.6 (9.1) | <0.001 |
| Residency, % (SD) |  |  |  |  |
| California | 95.9 (6.0) | 95.3 (6.8) | 97.0 (4.3) | <0.001 |
| Out of State/International | 1.7 (2.7) | 1.7 (2.9) | 1.7 (2.1) | 0.836 |
| Unknown/ Homeless | 2.1 (4.4) | 2.7 (4.7) | 1.1 (3.8) | <0.001 |
| Spoken language, % (SD) |  |  |  |  |
| English | 85.0 (11.5) | 85.6 (11.5) | 84.1 (11.4) | <0.001 |
| Other | 14.9 (11.5) | 14.3 (11.5) | 15.8 (11.4) | <0.001 |
| Disposition (%) |  |  |  |  |
| Routine care | 91.6 (5.2) | 91.4 (5.8) | 91.9 (4.2) | 0.003 |
| Other | 8.3 (5.2) | 8.5 (5.8) | 8.0 (4.2) | 0.003 |

Table displays volume and percentage share of mean quarterly visits to emergency departments of 281 general acute care hospitals in California before and during the unwinding period. Standard deviations are in parentheses. **a** The share of Invalid/Unknown observations is excluded. **b** Includes Other Non-federal Programs, Champus (Tricare), Disability, Other Federal Programs, Title V, Veterans Affairs Plans, Other Government, or Workers’ Compensation.

**Table 2A** Descriptive characteristics of ED visits by facility type in California, pre- and during Medicaid unwinding

|  | Mean Quarterly ED Visits | | | |
| --- | --- | --- | --- | --- |
|  | Total | Pre-unwinding | During unwinding | P-value |
|  | 2021Q1 to 20234Q4 | 2021Q1 to 2023Q2 | 2023Q3 to 2024Q4 |  |
| Total visits | 44,620,104 | 26,595,680 | 18,024,424 |  |
| Rurality % (SD) |  |  |  |  |
| Rural (n =52) | 8.0 (0.15) | 8.1 (0.14) | 8.0 (0.11) | <0.001 |
| Urban (n =229) | 92.0 (0.15) | 91.9 (0.14) | 92.0 (0.11) | <0.001 |
| Ownership % (SD) |  |  |  |  |
| State/local (n =50) | 13.8 (0.25) | 13.8 (0.31) | 13.8 (0.09) | 0.018 |
| Non-profit (n =165) | 69.3 (0.29) | 69.3 (0.37) | 69.3 (0.06) | 0.106 |
| Investor-owned (n =66) | 16.9 (0.21) | 16.9 (0.24) | 16.9 (0.12) | 0.051 |
| Bed-Size % (SD) |  |  |  |  |
| Less than 100 (n =60) | 9.3 (0.14) | 9.3 (0.14) | 9.2 (0.14) | <0.001 |
| 100-299 (n =141) | 46.4 (0.41) | 46.4 (0.48) | 46.3 (0.23) | <0.001 |
| 300 and more (n =80) | 44.3 (0.49) | 44.3 (0.56) | 44.4 (0.31) | <0.001 |
| Poverty level of counties % (SD) |  |  |  |  |
| Above mean (n =134) | 49.4 (0.48) | 49.4 (0.57) | 49.2 (0.19) | <0.001 |
| Below mean (n =147) | 50.6 (0.48) | 50.6 (0.57) | 50.8 (0.19) | <0.001 |

Table displays volume and percentage share of mean quarterly visits to emergency departments of 281 general acute care hospitals in California before and during the unwinding period. Standard deviations are in parentheses.

**Table 3A** Association of Medicaid Unwinding with Quarterly Payer Mix of Outpatient ED Visits in California (2021–2024), Weighted and Adjusted Estimates, Balanced Sample **^a^**

|  | Pre-unwinding trend | Level change after unwinding | Trend change relative to the pre-unwinding trend |
| --- | --- | --- | --- |
| Medi-Cal | 0.27*** | -0.23*** | -0.32*** |
| Private | 0.08*** | -0.47*** | -0.13*** |
| Uninsured | -0.06*** | 0.07*** | 0.11*** |
| Medicare | -0.08*** | 0.15*** | 0.12*** |
| Other | -0.04*** | 0.12*** | 0.07*** |

**a** The estimates are for 281 facilities licensed as general acute care hospital. Each row presents estimates from a separate ITS regression model stratified by payer type. Coefficients for trends and level-changes are based on weighted interrupted time-series estimates accounting for the visit-volume of each facility. The models are adjusted for patient characteristics (e.g., age, race and ethnicity, sex, language, and disposition), facility fixed effects, and quarter fixed effects. Robust standard errors clustered at facility level are in parentheses. Asterisks in the table indicate the statistical significance of the results, with p < 0.05 (*), p < 0.01 (**), and p < 0.001 (***).

**Table 4A** Association of Medicaid Unwinding with Quarterly Payer Mix of Outpatient ED Visits in California (2021–2024), Adjusted Estimates, Full Sample **^a^**

|  | Pre-unwinding trend | Level change after unwinding | Trend change relative to the pre-unwinding trend |
| --- | --- | --- | --- |
| Medi-Cal | 0.27***  (0.06) | -0.10  (0.26) | -0.46***  (0.09) |
| Private | 0.12*  (0.05) | -0.63***  (0.19) | -0.17*  (0.09) |
| Uninsured | -0.11***  (0.03) | 0.28  (0.17) | 0.17***  (0.04) |
| Medicare | -0.11***  (0.02) | 0.10  (0.09) | 0.21***  (0.05) |
| Other | -0.04*  (0.02) | 0.09*  (0.05) | 0.07**  (0.02) |

**a** The estimates are for 309 facilities licensed as general acute care hospital. Coefficients for trends and level-changes are based on unweighted interrupted time-series estimates. The models are adjusted for patient characteristics (e.g., age, race and ethnicity, sex, language, and disposition), facility fixed effects, and quarter fixed effects. Robust standard errors clustered at facility level are in parentheses. Asterisks in the table indicate the statistical significance of the results, with p < 0.05 (*), p < 0.01 (**), and p < 0.001 (***).

**Table 5A** Association of Medicaid Unwinding with Quarterly Payer Mix of Outpatient ED Visits in California (2021–2024), Adjusted Estimates, Subcategory of Hospitals **^a^**

|  | Pre-unwinding trend | Level change after unwinding | Trend change relative to the pre-unwinding trend |
| --- | --- | --- | --- |
| **Rurality** | | | |
| Rural hospitals |  |  |  |
| Medi-Cal (mean=41.6) | 0.10  (0.10) | -0.16  (0.33) | -0.41  (0.24) |
| Private (mean=25.3) | 0.25*  (0.13) | -0.37  (0.36) | -0.13  (0.20) |
| Uninsured (mean=4.8) | -0.19***  (0.03) | 0.50***  (0.14) | 0.29***  (0.06) |
| Medicare (mean=23.6) | -0.15*  (0.06) | 0.25  (0.24) | 0.16  (0.13) |
| Other (mean=4.4) | 0.01  (0.02) | 0.01  (0.13) | 0.06  (0.03) |
| Urban hospitals |  |  |  |
| Medi-Cal (mean=41.7) | 0.24***  (0.05) | 0.02  (0.26) | -0.38***  (0.07) |
| Private (mean=29.6) | 0.10**  (0.03) | -0.57***  (0.15) | -0.12*  (0.06) |
| Uninsured (mean=5.7) | -0.07*  (0.03) | 0.15  (0.21) | 0.12**  (0.04) |
| Medicare (mean=19.5) | -0.08***  (0.02) | -0.05  (0.10) | 0.17***  (0.05) |
| Other (mean=3.0) | -0.06**  (0.02) | 0.14**  (0.05) | 0.07*  (0.03) |
| **Ownership** | | | |
| State/local-owned hospitals |  |  |  |
| Medi-Cal (mean=43.2) | 0.10  (011) | -1.1**  (0.39) | -0.21  (0.27) |
| Private (mean=27.0) | 0.32**  (0.12) | -0.36  (0.34) | -0.39  (0.20) |
| Uninsured (mean=5.6) | -0.13**  (0.05) | 0.50*  (0.25) | 0.22**  (0.08) |
| Medicare (mean=19.4) | -0.07  (0,03) | 0.29  (0.26) | 0.20  (0.16) |
| Other (mean=4.2) | -0.02  (0.03) | 0.07  (0.12) | 0.03  (0.06) |
| Non-Profit hospitals |  |  |  |
| Medi-Cal (mean=39.1) | 0.16**  (0.05) | 0.08  (0.32) | -0.18**  (0.06) |
| Private (mean=30.2) | 0.08**  (0.03) | -0.23*  (0.12) | -0.09*  (0.04) |
| Uninsured (mean=4.9) | -0.08*  (0.04) | 0.34  (0.24) | 0.11**  (0.03) |
| Medicare (mean=22.3) | -0.09***  (0.02) | 0.17*  (0.07) | 0.07**  (0.02) |
| Other (mean=3.2) | -0.04  (0.02) | 0.07  (0.05) | 0.05  (0.03) |
| Investor-owned hospitals |  |  |  |
| Medi-Cal (mean=47.2) | 0.28**  (0.10) | 0.24  (0.33) | -0.87***  (0.16) |
| Private (mean=26.9) | -0.07  (0.09) | -0.16  (0.29) | 0.18  (0.15) |
| Uninsured (mean=7.3) | -0.12**  (0.03) | 0.33*  (0.16) | 0.14*  (0.06) |
| Medicare (mean=15.9) | -0.04  (0.03) | -0.77*  (0.33) | 0.31*  (0.12) |
| Other (mean=2.6) | -0.02  (0.01) | -0.01  (0.06) | 0.04  (0.02) |
| **Bed Size** | | | |
| Less than 100 beds |  |  |  |
| Medi-Cal (mean=39.8) | 0.13  (0.09) | -0.38  (0.33) | -0.26  (0.22) |
| Private (mean=27.1) | 0.26*  (0.11) | -0.56  (0.31) | -0.18  (0.17) |
| Uninsured (mean=4.8) | -0.16***  (0.03) | 0.45**  (0.14) | 0.22***  (0.05) |
| Medicare (mean=23.9) | -0.14*  (0.06) | 0.24  (0.26) | 0.11  (0.12) |
| Other (mean=4.3) | -0.02  (0.02) | 0.11  (0.06) | 0.06*  (0.03) |
| 100-299 beds |  |  |  |
| Medi-Cal (mean=40.2) | 0.19***  (0.04) | -0.26  (0.16) | -0.47***  (0.08) |
| Private (mean=31.1) | 0.09*  (0.04) | -0.62**  (0.18) | -0.10  (0.08) |
| Uninsured (mean=5.7) | -0.10***  (0.02) | 0.46***  (0.10) | 0.16***  (0.04) |
| Medicare (mean=19.6) | -0.05*  (0.02) | -0.18  (0.13) | 0.19***  (0.06) |
| Other (mean=2.9) | -0.03  (0.02) | 0.06  (0.07) | 0.03  (0.03) |
| 300 beds and more |  |  |  |
| Medi-Cal (mean=46.0) | 0.33***  (0.07) | 0.07  (0.64) | -0.32*  (0.15) |
| Private (mean=26.1) | 0.01  (0.03) | -0.19  (0.20) | -0.02  (0.06) |
| Uninsured (mean=5.8) | -0.02  (0.07) | -0.25  (0.51) | 0.06  (0.08) |
| Medicare (mean=18.7) | -0.08**  (0.02) | 0.05  (0.14) | 0.10*  (0.05) |
| Other (mean=3.0) | -0.06  (0.04) | 0.06  (0.04) | 0.07  (0.05) |
| **Poverty Level of Hospital Counties** | | | |
| High poverty rate |  |  |  |
| Medi-Cal (mean=44.7) | 0.14  (0.07) | 0.23  (0.37) | -0.49***  (0.13) |
| Private (mean=27.0) | 0.13  (0.08) | -0.33  (0.22) | -0.07  (0.10) |
| Uninsured (mean=5.6) | -0.13**  (0.04) | 0.09  (0.29) | 0.24***  (0.04) |
| Medicare (mean=19.4) | -0.12***  (0.03) | 0.08  (0.17) | 0.27**  (0.08) |
| Other (mean=3.0) | -0.04  (0.03) | 0.04  (0.05) | 0.07*  (0.03) |
| Low poverty rate |  |  |  |
| Medi-Cal (mean=38.4) | 0.24***  (0.05) | -0.67***  (0.17) | -0.25***  (0.06) |
| Private (mean=30.9) | 0.06  (0.04) | -0.33*  (0.14) | -0.09  (0.05) |
| Uninsured (mean=5.5) | -0.08***  (0.02) | 0.37***  (0.10) | 0.10***  (0.03) |
| Medicare (mean=21.3) | -0.07***  (0.02) | 0.03  (0.09) | 0.06  (0.03) |
| Other (mean=3.5) | -0.05**  (0.02) | 0.10  (0.07) | 0.05  (0.03) |

**a** The estimates are for subcategory of 281 facilities licensed as general acute care hospital. Each row presents estimates from a separate ITS regression model stratified by hospital subgroup and payer type. Coefficients for trends and level-changes are based on unweighted interrupted time-series estimates. The models are adjusted for patient characteristics (e.g., age, race and ethnicity, sex, language, and disposition), facility fixed effects, and quarter fixed effects. Robust standard errors clustered at facility level are in parentheses. Asterisks in the table indicate the statistical significance of the results, with p < 0.05 (*), p < 0.01 (**), and p < 0.001 (***).


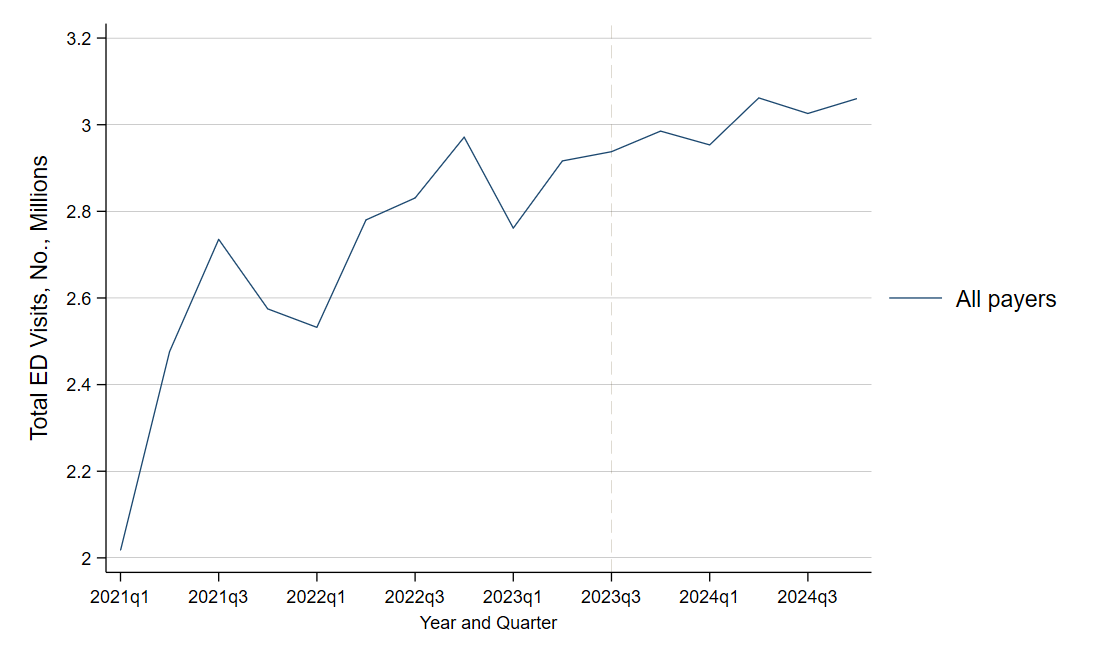
**Figure 1A**: Trends In the Quarterly Number of All Outpatient Emergency Department (ED) Visits in California, 2021-2024


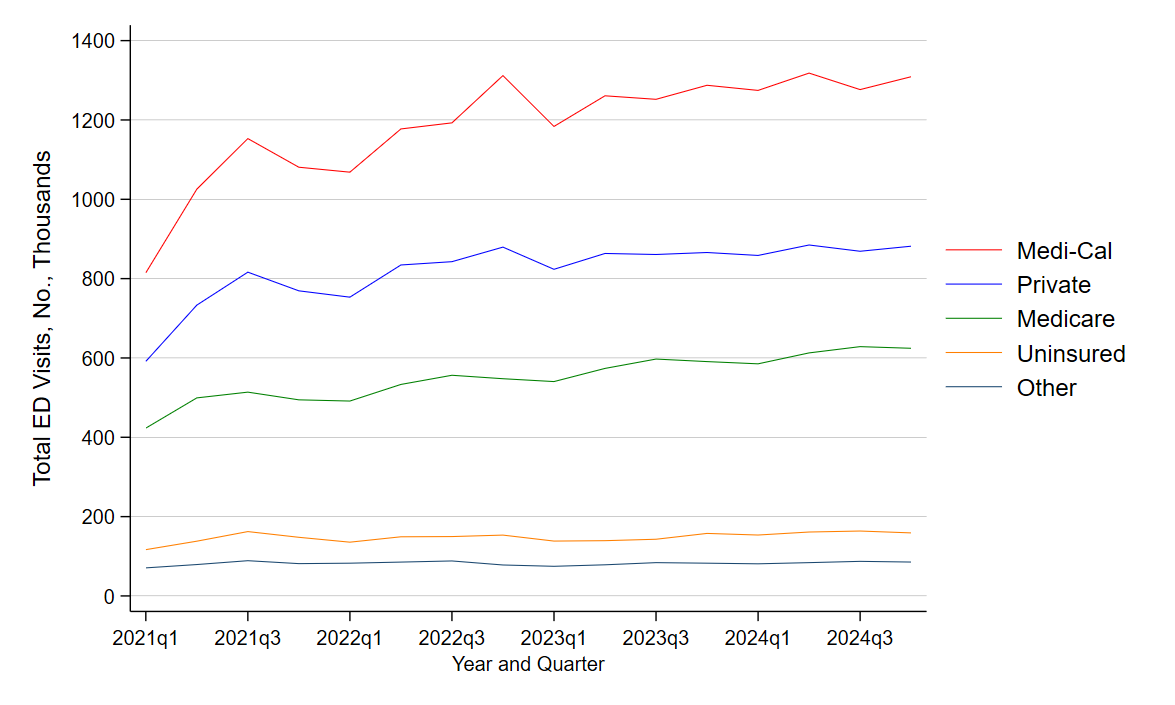


**Figure 2A**: Trends In the Quarterly Number of All Outpatient Emergency Department (ED) Visits by Payers in California, 2021 To 2024

Estimation of additional cost of uninsured visits due to unwinding

**Inputs**

Number of facilities=281

Count of quarters during the unwinding=6

Total visit​s (over 6 quarters during unwinding) = 18,024,424

Total uninsured visits (over 6 quarters during unwinding) = 938,000

Increase in uninsured visits (over 6 quarters during unwinding) = 0.96%

Average cost of treat-and-release ED visits for routine discharges = $710

Medicaid reimbursement ratio compared to uninsured =40%

**Estimations**

Uninsured rate=938,000 ÷ 18,024,424=5.21%

Counterfactual uninsured rate=5.21% − 0.96%=4.25%

Counterfactual uninsured visits=0.25% × 18,024,424= 766,965

Additional uninsured visits= 938,000 - 766,965=171,000

Additional cost of uninsured visits=171,000 × $710= $121,410,000

Estimated revenue loss, total =60% × $121,410,000=$72,846,000

Estimated revenue loss per facility=$72,846,000 ÷ 281=$259,238

Estimated revenue loss, per facility per quarter=$259,238 ÷ 6=$43,206
